# Sinapic Acid Affects Phenolic and Trichothecene Profiles of *F. culmorum* and *F. graminearum* Sensu Stricto

**DOI:** 10.3390/toxins9090264

**Published:** 2017-08-28

**Authors:** Tomasz Kulik, Kinga Stuper-Szablewska, Katarzyna Bilska, Maciej Buśko, Anna Ostrowska-Kołodziejczak, Dariusz Załuski, Juliusz Perkowski

**Affiliations:** 1 Department of Botany and Nature Protection, University of Warmia and Mazury in Olsztyn, Plac Łódzki 1, 10-727 Olsztyn, Poland; katarzyna.bilska@uwm.edu.pl; 2 Department of Chemistry, Poznan University of Life Sciences, Wojska Polskiego 75, 60-637 Poznan, Poland; kstuper@up.poznan.pl (K.S.-S.); mabu@au.poznan.pl (M.B.); ostrowska.anna.maria@gmail.com (A.O.-K.); julperk@au.poznan.pl (J.P.); 3 Department of Plant Breeding and Seed Production, University of Warmia and Mazury in Olsztyn, Plac Łódzki 3, 10-727 Olsztyn, Poland; dariusz.zaluski@uwm.edu.pl

**Keywords:** *Fusarium*, trichothecenes, sinapic acid

## Abstract

Plant-derived compounds for reducing the mycotoxin load in food and feed have become a rapidly developing research field of importance for plant breeding efforts and in the search for natural fungicides. In this study, toxigenic strains of *Fusarium culmorum* and *F. graminearum* sensu stricto were exposed to sinapic acid on solid YES media at levels close to those reported in wheat bran. Fusaria produced phenolic acids, whose accumulation was decreased by exogenous sinapic acid. Strains exposed to the lowest doses of sinapic acid showed more efficient reduction of phenolic acid production than fungi kept at higher concentrations of this compound. Fungi reduced exogenous sinapic acid, leading to the formation of syringic aldehyde. Treatment with sinapic acid led to a dramatic accumulation of its parent compound ferulic acid, presumably due to inhibition of the further conversion of this phenolic compound. Exogenous sinapic acid decreased the production of trichothecenes by fungi. Higher doses of sinapic acid resulted in more efficient reduction of mycotoxin accumulation in the media. Gene expression studies of *Tri* genes responsible for trichothecene biosynthesis (*Tri4*, *Tri5* and *Tri10*) proved that the inhibition of mycotoxin production by sinapic acid occurred at the transcriptional level. Fusaria respond to sinapic acid by stimulation of ergosterol biosynthesis.

## 1. Introduction

Fusarium head blight (FHB) of small grain cereals is one of the most serious diseases affecting grain production worldwide. Among the predominating fungi causing the disease are *Fusarium culmorum* and *Fusarium graminearum* sensu stricto (s.s.). The latter appears to be responsible for the current global outbreaks of FHB [1,2]. In Europe, both species reduce yield and cause quality deterioration by contamination of the grain with type B trichothecenes, including deoxynivalenol (DON), nivalenol (NIV), and their acetylated derivatives: 3-acetyldeoxynivalenol (3ADON), 15-acetyldeoxynivalenol (15ADON), and 4-acetylnivalenol (4ANIV, syn. fusarenone-X) [3,4]. 

Fungicides, mainly belonging to the azole class, have been critical to control Fusaria in the field [5]. However, results on how azole treatment affects mycotoxin accumulation are contradictory [6], presumably due to the difficulty of timing and targeting fungicides to the ear at anthesis [7]. 

There has been increasing concern recently regarding the risk to human health and the environment which has prompted a search for new, efficient, environmentally-friendly and sustainable disease management strategies [8]. Previous studies have highlighted that natural and/or natural-like cinnamic-derived phenolic acids may pose a promising source for the development of novel inhibitors of trichothecene production by Fusaria [9,10]. 

In plants, these secondary metabolites contribute to disease resistance [11,12,13]. These metabolites have been found to reduce pathogen development through increased host cell wall thickening, as well as direct antifungal activity, which limits fungal growth [12]. The ability of phenolic acids to inhibit mycotoxin production has been linked to their antioxidant and antiradical properties [14]. This contributes to their potential of scavenging reactive oxygen species (ROS), which have been found to increase mycotoxin production by Fusaria both in vitro and *in planta* [15,16].

Cereal bran layers contain the highest levels of phenolic acids [17,18]. For example, in wheat, sinapic acid occurs mostly in aleurone (440 μg/g) and bran (250 μg/g), which is 44 to 25 fold higher than in endosperm [18]. 

Recent in vitro studies demonstrated the inhibitory activities of cinnamic-derived phenolic acids (ferulic, chlorogenic and *trans*-cinnamic acid) against trichothecene production by *F. culmorum* and *F. graminearum* s.s. [9,10,19,20]. However, no data is available regarding the response of fungi to sinapic acid, which is among the most common hydroxycinnamic acids widespread in the plant kingdom. Sinapic acid is formed as esters between different derivatives of *trans*-cinnamic and quinic acid molecules [21,22] and has been previously identified as a resistance biomarker metabolite in cereals against Fusaria [12]. Expressing both antioxidant and antibacterial effects, sinapic acid represents an interesting compound for consideration as a preservative in foods [23].

To date, however, studies on the effect of sinapic acid on fungi are still scarce and limited mainly to assessing its antifungal activity [24]. Other limited studies showed that the ascomycete fungi can transform exogenous sinapic acid to syringic acid via propenoic chain cleavage, which appears to be common in filamentous fungi [25,26]. A recent study showed that Fusaria can produce sinapic acid in vitro, comprising around 15% of the sum fungal-derived phenolic acids [20]. Production of phenolic acids appears to be common in fungi (including the genus *Fusarium*), which suggests that the ability to synthesize these secondary metabolites is important in the life cycle of fungi [20].

In this study, under in vitro conditions, strains of *F. culmorum* and *F. graminearum* s.s. were incubated at different levels of sinapic acid. Doses of sinapic acid used in the in vitro experiments were close to amounts reported in wheat bran [18]. The effect of treatment with this compound was assessed on fungal secondary metabolic profiles. It was found that treatment with sinapic acid affected the production of phenolic acids by fungi. The impact of exogenous sinapic acid on both trichothecene accumulation in the media and the expression of *Tri* genes (*Tri4*, *Tri5* and *Tri10*) responsible for trichothecene biosynthesis was also analyzed. It was also found that exogenous sinapic acid impacted the biosynthesis of ergosterol by fungi. These results not only broadened knowledge of the response of Fusaria to phenolic acids but also indicated that sinapic acid could have great potential for developing strategies aimed at limiting the mycotoxin load in food and feed.

## 2. Results

### 2.1. Exogenous Sinapic Acid Affects Phenolic Acids Profiles of F. culmorum and F. graminearum s.s.

Chemical analyses of YES+sinapic acid control plates, showed that the amounts of exogenous sinapic acid initially dissolved in ethanol were unaffected after a 21-day of incubation period (Supplementary File 1). Apart from sinapic acid, other tested phenolic acids were present in phenolic-acid-treated controls at levels close to those found in the YES-only control. 

Fungi exposed to either 100, 400 or 800 μg/g sinapic acid, significantly (*p* < 0.001) reduced this phenolic compound by 37–64%, 43–51% and 32–41%, respectively. Surprisingly, the reduction of sinapic acid was not linked to the formation of syringic acid. In a separate experiment, fungal cultures exposed to 800 μg/g sinapic acid were screened for the presence of syringic aldehyde, an intermediate molecule between sinapic and syringic acid. It was found that the concentration of syringic aldehyde in samples treated with sinapic acid dramatically increased compared to YES+fungal controls (Supplementary File 1), which demonstrates that the first step of the route of sinapic acid conversion is its oxidation to syringic aldehyde.

Interestingly, we found dramatic accumulation of ferulic acid, which is the parent intermediate of sinapic acid. Ferulic acid increased by 56–219% and 205–273% after treatment with 400 and 800 μg/g sinapic acid, respectively. 

The concentration of other phenolic acids (*trans*-cinnamic, caffeic, *p*-coumaric, syringic, gallic, gentisic and *p*-hydroxybenzoic acid) decreased. The exception was chlorogenic acid, which dramatically increased by 179–291% after treatment with 800 μg/g sinapic acid. 

Importantly, the total amount of phenolic acids was significantly lower in cultures treated with sinapic acid than in corresponding YES+fungal controls, which indicates inhibition of *PAL* (phenylalanine ammonia-lyase) expression by sinapic acid. However, this effect appears to be largely dependent on the sinapic acid dose assayed (Supplementary File 1). Strains exposed to the lowest doses of sinapic acid showed the most efficient reduction of phenolic acid production, which gradually increased with the dose of this exogenously applied compound.

### 2.2. Exogenous Sinapic Acid Lowers Trichothecene Accumulation in the Media

Fungi kept in the presence of sinapic acid decreased trichothecene accumulation in the media. The inhibitory effect of sinapic acid appeared to be largely dependent on its concentration and the assayed strain (Table 1). The higher dose of sinapic acid, the greater inhibition of mycotoxin accumulation in the media. Fungi kept at 100 μg/g sinapic acid displayed reduction in mycotoxin content by 30.4–95%, except for CBS 173.31 showing only 18% reduction of trichothecene content. A higher dose of sinapic acid (400 μg/g) reduced mycotoxin levels by 73.2–97.7%. The exception was CBS 173.31, which showed a 36.8% reduction of trichothecene accumulation. The strains incubated at the highest dose of sinapic acid (800 μg/g) showed the most dramatic reduction of mycotoxin accumulation by 88.1–99.7%.

### 2.3. Sinapic Acid Inhibits the Activity of Tri Genes Involved in Trichothecene Biosynthesis

The effect of sinapic acid on gene expression was evaluated by quantification of *Tri* transcripts of *Tri4*, *Tri5* and *Tri10* genes at day 3 of incubation (Table 1). Both *Tri5* and *Tri4* genes encode the first steps of the trichothecene biosynthesis pathway. The expression of both is regulated by *Tri10* [27,28]. Sinapic acid inhibited the activity of *Tri* genes. Only two fungal cultures (CBS 119173 and CBS 138561) supplemented with the lowest dose of sinapic acid (100 μg/g) showed no significant fold-change values [*P*(H1) = 0.001] of *Tri* transcript levels (Table 1). Higher doses of this assayed phenolic compound generally caused a corresponding decrease in *Tri* transcript levels. 

### 2.4. Sinapic Acid Stimulates Ergosterol Biosynthesis by Fungi

Ergosterol is the prevalent sterol of the fungal cell membrane [29]. It plays an important role in regulating membrane fluidity and plasma membrane biogenesis and function [30]. Supplementary File 1 shows the effect of sinapic acid on ergosterol production by Fusaria. It was found that the production of this membrane sterol was stimulated by sinapic acid, presumably as a response to the strong antifungal activity of this phenolic compound on fungal growth.

## 3. Discussion

The need for the discovery and development of novel disease management strategies to control Fusaria is nowadays of great importance due to the loss of existing products through the development of fungicide resistance and the desire for products with more favorable environmental impact [10,31]. The recent evidence from in vitro studies on the effect of phenolic acids on Fusaria provides the scientific framework for developing and adapting strategies incorporating the application of these compounds to reduce mycotoxin load in plant-derived foods [9,10,20]. 

To date, however, the mechanisms by which these compounds interfere with the biosynthesis of mycotoxins are not yet completely understood. One proposed hypothesis is that phenolic acids affect trichothecene biosynthesis at a transcriptional level [32]. Inhibition of the expression of *Tri* genes has been recently confirmed by *trans*-cinnamic acid, the precursor of the other phenylpropanoids [20]. Other studies have provided corresponding evidence on the mechanism of action of ferulic acid [9], exhibiting antioxidant and antiradical properties similar to sinapic acid (Table 2). Results of gene expression experiments obtained in this study support this view. It was found that sinapic acid inhibits trichothecene production by fungi by repressing *Tri* gene expression, regardless of the fungal chemotype.

Previous studies showed that exogenous phenolic acids such as chlorogenic [19,20] and *trans*-cinnamic acids [20] can be degraded or converted by Fusaria. However, to date, no data has been provided on the degradation/conversion of sinapic acid by these phytopathogens. Ascomycete soil fungi have been found to convert sinapic to syringic acid [25,26]. In this pathway, sinapic acid is first converted to syringic aldehyde, which is oxidized to syringic acid. In this study, it was found that the reduction of exogenous sinapic acid by Fusaria was associated with a dramatic increase of syringic aldehyde in the media. The conversion of syringic aldehyde to syringic acid was not detected, presumably due to the short incubation period of fungi on the media.

**Figure 1:**
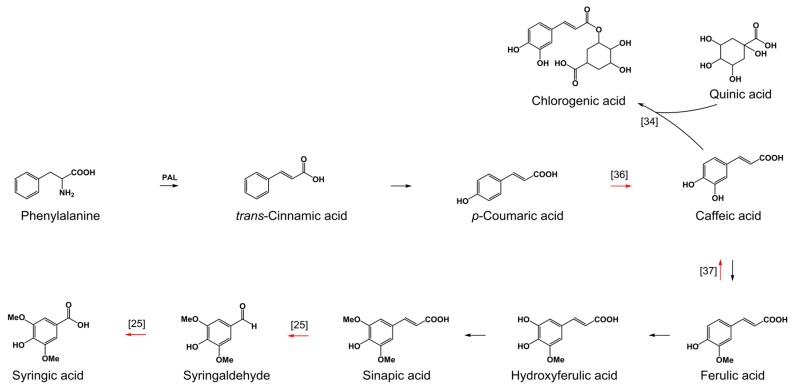
Schematic representation of some of the core biochemical pathways of major phenolic acids with an indication of the routes of the conversion and degradation of phenolic acids by fungi. Red arrows indicate the routes of either the conversion or degradation of phenolic acids by fungi. The numbers near the arrows indicate the literature from which the data originates [25,34,36,37].

Recently, it was shown that phenolic acids affect fungal-derived phenolic acid profiles [20]. The results presented in this paper support previous findings showing that treatment with phenolic acids decreases the accumulation of fungal-derived phenolic acids. However, it is unclear why treatment with lower amounts of phenolic acids is more efficient in suppressing the production of these secondary metabolites by fungi. It was found that treatment with sinapic acid led to dramatic accumulation of its parent compound ferulic acid, presumably due to inhibition of the conversion of this phenolic intermediate. Unexpectedly, besides ferulic acid, an increased accumulation of chlorogenic acid in fungal cultures treated with 800 μg/g sinapic acid was also found. Chlorogenic acid is formed by the esterification of caffeic and quinic acids [34]. It is hypothesized that a dramatic increase could be associated with increased accumulation of caffeic acid, the parent metabolite of ferulic acid (Figure 1). Caffeic acid may dramatically increase in the early incubation period of fungi as a consequence of increased accumulation of fungal-derived ferulic acid. Efficient conversion of caffeic acid might lead to the observed high accumulation of chlorogenic acid.

Phenolic acids display varying antifungal activities, which have been attributed to differences in their lipophilicity, scored using retention times [35]. Previous study suggested a reverse correlation between lipophilicity of phenolic acids and ergosterol production [20]. Lipophilicity of sinapic acid is close to its parent metabolite ferulic acid (Table 2), which has been demonstrated to exhibit strong antifungal properties [14]. In this study, it was shown that sinapic acid stimulates ergosterol production by fungi, which supports previous findings on overproduction of this membrane sterol in the presence of strong phenolic antifungals [20].

Overall, the present study showed for the first time that sinapic acid poses a promising source for the development of novel inhibitors of mycotoxin production by Fusaria. It is worth noting, however, that in vitro studies cannot consider a wide range of interfering factors such as bioavailability, biocompatibility and chemical compounds interactions. Long-term field trials need to be carried out in order to evaluate the efficacy of phenolic acids in controlling FHB and reducing mycotoxin contamination. Finally, environmental studies are also required to evaluate the impact of phenolic acids on field ecosystems.

## 4. Materials and Methods 

### 4.1. Fungal Strains

Six fungal strains were used in this study (Table 3). They were maintained in several international fungal collections: Westerdijk Fungal Biodiversity Institute, Utrecht, the Netherlands, MUCL—Mycothèque de l’Université catholique de Louvain, Louvain-la-Neuve, Belgium and ARS Culture Collection, USDA, Peoria, IL, USA. A detailed description of the fungal strains is given in ToxGen database [38].

### 4.2. Medium and Culture Conditions

Yeast extract-sucrose (YES) agar medium was used in this study. Sinapic acid (Sigma–Aldrich, Saint Louis, MO, USA) was dissolved in 10 mL of 96% ethanol and then added to YES medium to obtain the final concentrations: 100 μg/g, 400 and 800 μg/g. The study incorporated three different controls: YES-only control (YES medium only), YES+sinapic acid control (YES media supplemented with either 100, 400 or 800 μg/g of sinapic acid), and six YES+fungal controls (fungal strains incubated on YES media). 

Petri plates (Ø 80 mm) were inoculated at the centre with a 5-mm agar disc from 6–8-week-old laboratory stock cultures maintained at 4 °C on PDA slants and incubated at 25 °C (in triplicate) in the dark. The study included chemical and gene expression analyses. Chemical analyses incorporated determination of phenolic acids, trichothecenes and ergosterol in the media. For gene expression analysis, plates were incubated for three days for each condition, while for chemical analysis the plates were incubated for 21 days.

### 4.3. Determination of Phenolic Acids in the Medium

Fungal-derived phenolic compounds were determined in dried fungal cultures after a 21-day incubation period (Supplementary File 1) as previously described in Kulik et al. [20]. Syringic aldehyde was determined by UPLC similarly as phenolic acids described by Kulik et al. [20] with modifications. Chromatographic separation was performed on an Acquity UPLC Shield BEH 18 1.7 um 2.1 × 50 mm, with an acetonitrile 0.1% formic acid used as an elution phase (isocratic). The concentration of syringic aldehyde was determined using an internal standard at wavelengths λ = 320 nm. Aldehyde was identified based on a comparison of the retention time of the analyzed peak with the retention time of the standard, and by adding a specific amount of the standard to the analyzed samples and a repeated analysis. The detection level was 0.1 μg/g. The retention time of syringic aldehyde was 8.11 min.

### 4.4. Determination of Antioxidant Capacity (VCEAC/L) and Radical Scavenging Activity (ABTS) of Sinapic Acid

VCEAC/L and ABTS assays of phenolic acids were performed as previously described by Kim et al. [39]. And Re et al. [40], respectively.

### 4.5. Analysis of Trichothecenes from Fungal Cultures

Trichothecenes were determined in fungal cultures treated and non-treated (YES+fungal controls) with different concentrations of sinapic acid by GC-MS as previously described by Perkowski et al. [41] and Kulik et al. [42,43].

### 4.6. Extraction of Total RNA and Preparation of cDNA

The total RNA was extracted from 3-day-old fungal cultures from mycelium grown on YES medium treated and non-treated (YES+fungal controls) with sinapic acid. Six biological replications were prepared for each condition. Extraction of RNA and reverse-transcription were performed as previously described in Kulik et al. [42,43]. cDNA samples were stored at −25 °C for transcript quantification.

### 4.7. RT-qPCR and Data Analyses

*Tri4* and *Tri5* genes which are responsible for the initial stage in the trichothecene biosynthetic pathway were chose for RT-qPCR analysis, as previously described in Kulik et al. [42,43]. The molecular analysis also included the *Tri10* gene responsible for regulation of multiple *Tri* genes [27]. Probes, conjugated with an MGB group, were labeled at the 5′-end with FAM, while the *Ef1α* probe was labeled at the 5′-end with VIC. Primers were synthesized by Sigma-Aldrich (Saint Louis, MO, USA), while MGB probes were ordered from Life Technologies Oligos, Primers, Probes and Nucleotides Synthesis Service (Applied Biosystems, Foster City, CA, USA). Duplex RT-qPCR reaction conditions were used for each *Tri* transcript, including the *Ef1α* reference control as previously described in Kulik et al. [20,42,43]. The amplification efficiency of the assays was determined based on five 5-fold dilutions of the cDNA fungal template, and were: 99.7% (R^2^ = 0.957), 99.7% (R^2^ = 0.995) and 103% (R^2^ = 0.993) for *Tri4*, *Tri5* and *Tri10* assays, respectively. In this study, the relative quantification of *Tri* targets was normalized to an *Ef1α* reference gene. The Ct values of the target *Tri4*, *Tri5*, *Tri10* and reference *Ef1α* gene were compared in the control and treated samples and normalized relative to the Ct values obtained for the reference *EF1α* gene using the REST 2009 software [44].

### 4.8. Determination of Ergosterol

Ergosterol was first quantified in fungal cultures treated with sinapic acid and in six different YES+fungal controls using UPLC as described by Perkowski et al. [45]. Three biological replications were then examined for each condition. Ergosterol was also determined in the YES-only control and in YES+sinapic acid controls. In addition, antifungal activity of sinapic acid was recorded during the initial 5-day incubation period on the media as previously described by Kulik et al. [43].

### 4.9. Statistical Analyses

The significance of differences among mycotoxin levels was tested using Tukey’s HSD test (*p* < 0.05). The reduction of exogenous sinapic acid was tested using a single-sample *t*-Student test at *p* < 0.05. The impact of exogenously applied sinapic acid on the accumulation of other fungal-derived phenolic acids and ergosterol was tested using a *t*-Student test for independent groups with Cochran-Cox adjustment at *p* < 0.05. The inhibitory effect of sinapic acid on fungal growth was tested using Tukey’s HSD test (*p* < 0.05).

## Figures and Tables

**Table 1:** Trichothecene accumulation and RQ (relative quantification) of *Tri4*, *Tri5* and *Tri10* transcripts of fungal strains incubated in the presence of sinapic acid after a 21-day incubation period.

Sinapic Acid Level	Strain	Tri Genotype	Trichothecene Levels (mg/kg) (*n* = 3 in Each Condition)						RQ (*n* = 6 in Each Condition)		
			DON	3ADON	15ADON	NIV	4ANIV	Sum of Trichothecenes	*Tri4*	*Tri5*	*Tri10*
YES+fungal controls	MUCL 53469	3ADON	17.5 ± 0.4 (a)	23.1 ± 0.9 (a)				40.6			
	CBS 173.31	3ADON	63.7 ± 3.8 (a)	9.9 ± 0.4 (a)				73.6			
	CBS 139512	NIV				79.2 ± 3.2 (a)	96.4 ± 4.8 (a)	175.6			
	CBS 119173	3ADON	49.6 ± 3.6 (a)	12.2 ± 0.8 (a)				61.8			
	CBS 138561	15ADON	1.5 ± 0.7 (a)		1.5 ± 0.7			3			
	MUCL 53455	NIV				4.4 ± 0.2 (a)		4.4			
100 μg/g (0.45 mM)	MUCL 53469	3ADON	1.72 ± 0.04 (b)	7.75 ± 0.16 (b)				9.47	0.27 (0.22–0.34)	NS	NS
	CBS 173.31	3ADON	51.09 ± 1.02 (b)	9.27 ± 0.37 (a)				60.36	0.09 (0.06–0.15)	0.29 (0.25–0.35)	NS
	CBS 139512	NIV				55.44 ± 2.22 (b)	16.86 ± 0.84 (b)	72.3	0.15 (0.11–0.2)	NS	NS
	CBS 119173	3ADON	2.6 ± 0.2 (b)	0.42 ± 0.03 (c)				3.02	NS	NS	NS
	CBS 138561	15ADON	0.14 ± 0.01 (b)		ND			0.14	NS	NS	NS
	MUCL 53455	NIV				3.06 ± 0.18 (b)		3.06	0.224 (0.163–0.31)	0.554 (0.46–0.667)	0.241 (0.225–0.259)
400 μg/g (1.8 mM)	MUCL 53469	3ADON	0.37 ± 0.01 (c)	0.55 ± 0.03 (c)				0.92	0.08 (0.062–0.1)	0.06 (0.05–0.07)	0.25 (0.22–0.29)
	CBS 173.31	3ADON	41.21 ± 2.47 (c)	5.36 ± 0.22 (b)				46.57	0.12 (0.07–0.16)	0.17 (0.07–0.45)	0.37 (0.3–0.45)
	CBS 173.32	NIV				30.55 ± 0.92 (c)	16.54 ± 0.5 (b)	47.09	0.11 (0.08–0.18)	0.08 (0.04–0.23)	0.228 (0.12–0.45)
	CBS 173.33	3ADON	0.9 ± 0.05 (b)	1.5 ± 0.11 (b)				2.4	0.014 (0.012–0.017)	0.008 (0.004–0.015)	NS
	CBS 173.34	15ADON	0.08 ± 0,01 (b)		ND			0.08	0.001 (0–0.001)	0.009 (0.004–0.028)	NS
	CBS 173.35	NIV				0.35 ± 0.01 (c)		0.35	0.189 (0.14–0.271)	0.321 (0.234–0.455)	0.099 (0.092–0.106)
800 μg/g (3.6 mM)	MUCL 53469	3ADON	0.06 ± 0.01 (c)	0.44 ± 0.02 (c)				0.5	0.004 (0.003–0.006)	0.007 (0.05–0.01)	NS
	CBS 173.31	3ADON	0.22 ± 0.01 (d)	0.13 ± 0.003 (c)				0.35	0.02 (0.016–0.037)	0.07 (0.05–0.09)	0.27 (0.25–0.29)
	CBS 139512	NIV				13.82 ± 0.69 (d)	7.01 ± 0.14 (c)	20.83	0.003 (0.003–0.004)	0.01 (0.005–0.035)	NS
	CBS 119173	3ADON	2 ± 0.04 (b)	0.3 ± 0.01 (c)				2.3	0.001 (0.001)	0.001 (0.001)	0.195 (0,180–0.213)
	CBS 138561	15ADON	0.01 ± 0.01 (b)		ND			0.01	0.001 (0.001–0.001)	0.033 (0.015–0.062)	NS
	MUCL 53455	NIV				0.1 ± 0.003 (c)		0.1	0.048 (0.035–0.067)	0.082 (0.068–0.099)	0.039 (0.037–0.042)

Degree of inhibition: <25%; 25–50%; 50–75%; >75%. (a), (b), (c) letters indicate homogenous groups at *p* < 0.05 followed by the Tukey test. NS—not significant. ND—not detected.

**Table 2:** Retention times (min), antioxidant capacity (VCEAC/L) and radical scavenging activity (ABTS) of phenolic acids.

Phenolic Acid	Retention Times (Min) *	VCEAC/L	ABTS (μmol TROLOX/100 g s.m.)
Sinapic acid	7.2	121	194.5
Ferulic acid	7.3	88.3	117.6
*trans*-Cinnamic acid	9.6	812.3	314.9

*—from Labronici Bertin et al. [33].

**Table 3:** List of fungal isolates used in this study.

Species	Strain	Trichothecene Genotype	Origin, Host and Year of Isolation
*F. culmorum*	CBS 173.31, NRRL 26853	3ADON	Canada, oat, 1927
	MUCL 53469	3ADON	Belgium, corn, 2007
	CBS 139512	NIV	Poland, wheat kernel, 2003
*F. graminearum* s.s.	CBS 119173, NRRL 38369	3ADON	USA, Louisiana, wheat head, 2005
	CBS 138561	15ADON	Poland, wheat kernel, 2010
	MUCL 53455	NIV	Belgium, corn, 2007

## Supplementary Materials

The following are available online at www.mdpi.com/2072-6651/9/9/264/s1, Supplementary file 1: Phenolic acid and ergosterol accumulation after 21 days of incubation of fungi.
